# Loss of Let-7 MicroRNA Upregulates IL-6 in Bone Marrow-Derived Mesenchymal Stem Cells Triggering a Reactive Stromal Response to Prostate Cancer

**DOI:** 10.1371/journal.pone.0071637

**Published:** 2013-08-19

**Authors:** Shian-Ying Sung, Chia-Hui Liao, Hsun-Pai Wu, Wan-Chi Hsiao, I-Hui Wu, Jinpu, Yu, Sue-Hwa Lin, Chia-Ling Hsieh

**Affiliations:** 1 Graduate Institute of Cancer Biology, China Medical University, Taichung, Taiwan; 2 Center for Molecular Medicine, China Medical University Hospital, Taichung, Taiwan; 3 School of Medicine, China Medical University, Taichung, Taiwan; 4 Immunology and Cancer Biotherapy, Tianjin Medical University Cancer Institution and Hospital, Tianjin, China; 5 Department of Molecular Pathology, The University of Texas MD Anderson Cancer Center, Houston, Texas, United States of America; 6 Department of Biotechnology, Asia University, Wufeng, Taichung, Taiwan; 7 The Institute for Translational Medicine, Taipei Medical University, Taipei, Taiwan; Chang Gung University, Taiwan

## Abstract

Bone marrow-derived mesenchymal stem cells (MSCs) are able to migrate to tumors, where they promote tumorigenesis and cancer metastasis. However, the molecular phenotype of the recruited MSCs at the tumor microenvironment and the genetic programs underlying their role in cancer progression remains largely unknown. By using a three-dimensional rotary wall vessel coculture system in which human MSCs were grown alone or in close contact with LNCaP, C4-2 or PC3 prostate cancer cell lines, we established *in* *vitro* matched pairs of normal and cancer-associated MSC derivatives to study the stromal response of MSCs to prostate cancer. We observed that prostate cancer-associated MSCs acquired a higher potential for adipogenic differentiation and exhibited a stronger ability to promote prostate cancer cell migration and invasion compared with normal MSCs both *in vitro* and in experimental animal models. The enhanced adipogenesis and the pro-metastatic properties were conferred by the high levels of IL-6 secretion by cancer-associated MSCs and were reversible by functionally inhibiting of IL-6. We also found that IL-6 is a direct target gene for the let-7 microRNA, which was downregulated in cancer-associated MSCs. The overexpression of let-7 via the transfection of let-7 precursors decreased IL-6 expression and repressed the adipogenic potential and metastasis-promoting activity of cancer-associated MSCs, which was consistent with the inhibition of IL-6 3′UTR luciferase activity. Conversely, the treatment of normal MSCs with let-7 inhibitors resulted in effects similar to those seen with IL-6. Taken together, our data demonstrated that MSCs co-evolve with prostate cancer cells in the tumor microenvironment, and the downregulation of let-7 by cancer-associated MSCs upregulates IL-6 expression. This upregulation triggers adipogenesis and facilitates prostate cancer progression. These findings not only provide key insights into the molecular basis of tumor-stroma interactions but also pave the way for new treatments for metastatic prostate cancer.

## Introduction

Bone is the second most common site of human cancer metastasis [1], and also contributes directly to prostate cancer mortality and morbidity, with more than 85% of patients who die from prostate cancer have bone metastases [2], [3]. The quality of life of prostate cancer patients can be significantly compromised by skeletal metastases through the development of bone pain, cancer-associated bone fractures and spinal compression, bone-metastasis-evoked cranial neuropathy from base of skull syndromes, anemia and infection [4], [5]. In spite of the severe complications of prostate cancer skeletal metastasis, there have been few advances in the therapeutic arena to prevent or diminish these lesions [6]. It is critical that a solid understanding of the pathophysiology of the prostate cancer skeletal metastatic process is developed to provide the basis for creating strategies to prevent or diminish their occurrence and associated complications.

Research has provided evidence that tumor-microenvironment interactions are crucial in oncogenesis and cancer progression, as first described in 1889 by Paget who proposed that the seeding of metastatic cancer cells depends on the host organ microenvironment (the “seed and soil” concept) [7]. Although most host cells in the stroma possess certain tumor-suppressing abilities, the progression of carcinomas to high-grade malignancies is accompanied by profound histological changes in the tumor-associated stroma. These changes include stromal cell phenotypic switching, extracellular matrix remodeling and angiogenesis induction [8], [9]. The development of an altered stromal microenvironment in response to carcinoma is a common feature of many tumors and is likely to promote tumorigenesis. During the prostate cancer invasion process, for example, cancer epithelial cells have the capacity to promote the so-called “reactive” stroma response via the transdifferentiation of normal fibroblasts to the reactive myofibroblast phenotype. Unlike normal fibroblasts, reactive myofibroblasts drive further genetic and gene expression changes in prostate cancer cells, allowing for the growth and survival of the tumor and dissemination to distant organs with lethal effects [10]–[13]. Gene expression profiling of clinical specimens revealed concurrent and independent genetic alterations in the stromal and cancer epithelial cells [14], [15], confirming the co-evolution of cancer and stromal cellular responses. Clinicopathological studies have also proven a critical role for the reactive stroma in the postoperative outcome of patients [16]–[18]. The intricate intercellular communication between epithelial and stromal elements suggests the importance of epigenetic pathways in the facilitation of prostate cancer progression rather than a direct process simply attributed to cancer cells alone.

In mouse models as well as in humans have reported that tumor stromal cells can be derived from bone marrow-derived progenitor cells which can be mobilized into the circulation, migrate towards tumors, incorporate into the tumor microenvironment, and contribute to the growth of various tumors [19]–[21]. Bone marrow-derived mesenchymal stem cells (MSCs) are multipotent mesenchymal precursor cells that contribute to the maintenance and regeneration of a variety of connective tissues, including bone, adipose, cartilage, and muscle [22]. Recently, circulating MSCs have been shown to integrate into and persist in the tumor stroma [23], providing a novel platform for selective *in vivo* delivery of anticancer agents to invasive and metastasis tumors [24]–[27]. The interactions between MSCs and tumor cells are not limited to homing but also seem to induce more adverse effects. Many observations indicate that, in the tumor microenvironment, MSCs have several tumor growth promoting functions, including the expression of growth factors [28], promotion of tumor vessel formation [29] and creation of cancer stem cell niches [30]. However, the molecular phenotype of the recruited MSCs at the tumor microenvironment and the genetic programs underlying their property in cancer progression remains mainly unknown. Understanding how newly recruited MSCs are altered during tumor progression and how they reciprocally influence neoplastic progression may lead to the development of novel therapies aimed at reducing metastasis formation.

In the present study, we focused on defining if bone marrow MSCs “co-evolve” with prostate cancer epithelial cells through reciprocal tumor-stromal interaction. The role and specific molecular mechanisms of the reactive MSCs in prostate cancer progression was also elucidated.

## Materials and Methods

### Ethics Statement

The animal studies were carried out in strict accordance with the recommendations in the Guide for the Care and Use of Laboratory Animals of the National Institutes of Health. The protocol was approved by the Institutional Animal Care and Use Committee of China Medical University (IACUC# 101-76-N). All surgery was performed under Zoletil (tiletamine-zolazepam) anesthesia at a dose of 20 mg/kg by intraperitoneal injection. All efforts were made to minimize suffering.

### Cell Lines and Cell Culture

All cell culture media and reagents were purchased from Invitrogen (Carlsbad, CA). The prostate cancer cell lines LNCaP, C4-2 and PC3 were used in previous studies [13], [31] and grown in T medium supplemented with 5% fetal bovine serum (FBS). Human bone marrow-derived MSCs (hMSCs) and normal human prostate epithelial cells (PrEC) purchased from Lonza (Rockland, ME) were maintained in MSCGM™ and PrEGM™, respectively, according to the manufacturer’s instructions (Lonza). The 3A6 human bone marrow-derived MSC cell line [32] was maintained in DMEM-LG medium supplemented with 10% FBS. For three-dimensional (3-D) coculture, 1×10^7^ 3A6 cells were grown alone or mixed with equal numbers of LNCaP, C4-2 or PC3 cells in a rotary wall vessel (RWV) systems (Synthecon, Houston, TX) following previously established protocol [33] with minor modification. Briefly, the single cell suspension was added to vessels which were then filled with 10 ml medium composed of T medium and DMEM-LG (1∶1). The rotation of the vessels was initially set at 15–20 rpm and then increased to maintain cell aggregates in free suspension. The medium was changed every 3 days. To isolate 3A6 cells from 3-D chimeric tumoroids, cell aggregates were harvested from the RWV and disassociated with 0.25% trypsin after 2 weeks of coculture. 3A6 cells were selected by antibiotic selection with 800 µg/ml G418 for 1 week.

### Differentiation of MSCs

3A6 MSC cells were seeded in 6-well plates at a density of 10^4^ cells/cm^2^ for the induction of osteogenic and adipogenic differentiation under specific culture conditions. For osteogenic differentiation, cells were grown in osteogenic differentiation medium composed of DMEM-LG with 10% FBS, 10 nM dexamethasone, 50 µg/ml ascorbic acid, and 10 mM β-glycerophosphate for 14 days and then subjected to Alizarin Red S staining, as previously described [32]. For adipogenic differentiation, cells were cultured in adipogenic differentiation medium composed of DMEM-LG supplemented with 10% FBS, 100 nM dexamethasone, 0.45 mM 3-isobutyl-1-methylxanthine, 10 µg/ml insulin, and 50 µg/ml indomethacin for 21 days, and then subjected to Oil Red O staining. The formation of adipocytes was monitored by the appearance of lipid droplets under a microscope. To quantify staining, Oil Red-O was extracted with isopropanol containing 4% Nonidet P-40 and measured as absorbance at a wavelength of 510 nm. All chemicals were purchased from Sigma-Aldrich (St. Louis, MO).

### Real-time Quantitative PCR

Total cellular RNA and enriched small RNA species containing microRNA (miRNA) were isolated from cultured cells using the mirVana miRNA Isolation Kit (Ambion, Austin, TX) following the manufacturer’s protocol. cDNA was synthesized from enriched small RNA and total RNA annealed with stem-loop reverse transcriptional (RT) primers and random primers, respectively, and reverse transcribed with MMLV reverse transcriptase (Invitrogen). Quantitative PCR was performed using the LightCycler 480 TaqMan master kit with miRNA- or gene-specific primers and the corresponding Universal Probe Library probe (Roche Applied Science, Mannheim, Germany). The stem-loop RT primers, the PCR primers, and probes were designed and sequences are shown in Table S1. The real-time PCR reaction was conducted according to the manufacturer’s instructions and was described in a previous study [34]. U6 small nuclear RNA and heat shock 90 kD protein 1, beta (HSPCB) were used as housekeeping genes for normalizing the expression of each miRNA and gene, respectively.

### Cytokine Antibody Array and ELISA

The expression levels of various cytokines in conditioned medium were measured using a Human Cytokine Antibody Array C Series 2000 kit (RayBiotech, Norcross, GA) as previously described [27]. Human interleukin 6 (IL-6) protein levels in conditioned medium were quantified using commercial ELISA kits (eBioscience, San Diego, CA) according to the manufacturer’s instructions.

### Oligonucleotide Transfection

3A6 cells were seeded at 2×10^5^ cells/well in 6-well plates and incubated overnight. Cells were transfected with either pre-miRNAs (pre-miR negative control and pre-miR-let-7c) (Ambion, Austin, TX) or the LNA™ miRNA inhibitors (anti-miR negative control and anti-miR-let-7) (Exiqon, Vedbaek, Denmark) at a final concentration of 10 nM or 30 nM, respectively, using DharmaFECT transfection reagent (Dharmacon, Lafayette, CO) according to the manufacturer’s instructions.

### Reporter Vectors and Luciferase Assay

The oligonucleotides of the putative let-7c recognition element, either the wild-type or the mutant sequence, at nucleotides 316–322 of the 3′-untranslated region (3′-UTR) of the human IL-6 gene were designed with flanking HindIII and SpeI sites and synthesized. After annealing the sense and antisense oligonucleotides, the resultant DNA fragments were cloned into the pMir-REPORT-Luc vector (Ambion) after HindIII and SpeI digestion. The resultant pMir-REPORT-Luc plasmids were mixed with pCMV-β-gal (galactosidase) at a 5∶1 molar ratio and were co-transfected with the pre-miRNAs into HEK 293 cells. After 24 h of incubation, the cell extracts were prepared for luciferase and β-gal activity assays using the Luciferase Assay System and β-Galactosidase Enzyme Assay System (Promega, Madison, WI). The relative luciferase activity was calculated by dividing the luciferase relative light units by the corresponding value for β-gal activity present in each sample.

### *In vitro* Migration and Invasion Assay

hMSCs or 3A6 derivatives (2×10^5^ cells) were grown in the lower chamber of transwells with serum-free RPMI-1640 (Invitrogen). After 24 h of incubation, 2×10^5^ prostate cancer cells in serum-free RPMI-1640 were added to the upper chamber containing an 8-µm-pore polycarbonate filter which was coated with (invasion) or without (migration) Matrigel (Becton Dickinson, Franklin Lakes, NJ). After 16 h incubation, cells on the lower surface of the membrane were stained with crystal violet and counted under a Zeiss Axiovert 200 fluorescent microscope (Carl Zeiss MicroImaging, Thornwood, NY) with a 10x objective.

### Animal Studies

Six-week-old male nude mice (BALB/cAnN.Cg-Foxn1nu/CrlNarl) were obtained from the National Laboratory Animal Center (Taipei, Taiwan). 1×10^6^ PC3 cells, either alone or mixed with equal number of 3A6^RWV^ or 3A6^PC3^ cells in 100 µl of PBS, were subcutaneously injected into the flanks of the mice (*n = *10 in each group). Tumor volume measurements were taken weekly. Mice were euthanized 10 weeks after cell inoculation, and tumors were excited for histopathologic examination.

### Immunohistochemistry

Specific protein was detected in tumor sections using a Novolink Polymer Detection System (Leica Microsystems, Newcastle Upon Tyne, UK) following the kit instructions as described previously [35]. The mouse monoclonal antibodies against ki-67 and CD31 were purchased from Leica Microsystems and diluted 1∶100 and 1∶200, respectively. To detect lipids, tissue slides were dehydrated with absolute propylene glycol for 5 min and stained with 0.5% Oil red O solution for 48 h. Tissue sections were counter-stained with hematoxylin.

### Statistical Analysis

All data are presented as the mean ± SD unless otherwise specified. Differences between groups were analyzed using the two-tailed Student’s *t*-test or one-way ANOVA for multiple comparisons. A *p*-value less than 0.05 was defined as statistically significant.

## Results

### Establishment of Matched Pairs of Normal and Cancer-associated MSC from Chimeric Prostate Tumoroids Under 3-D RWV Conditions

**Figure 1:**
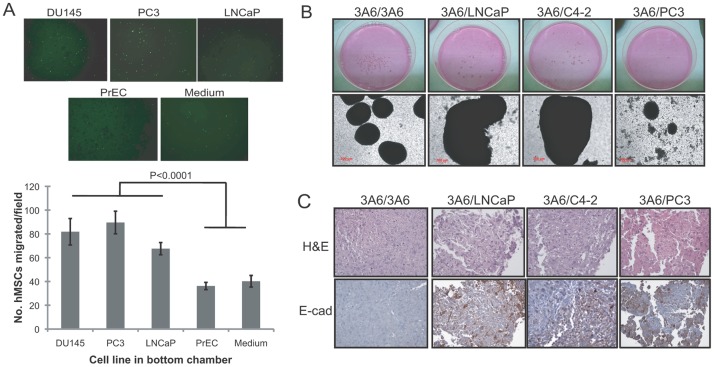
Interactions of human bone marrow-derived MSCs and prostate cancer cells. (A) Migratory response of human MSCs to the medium, normal prostatic epithelial cells (PrEC) and prostate cancer cell lines (DU145, PC3 and LNCaP). Migrated cells through the membrane of transwells were stained with crystal violet 8 h after cell plating. The lower surface of the membrane was photographed (100x), and a representative image from each condition is shown at top. Quantitative data are represented as the means ± SD of the number of cells per high-power field (100x) in triplicate experiments. (B) Macroscopic (upper) and microscopic (bottom) view of cellular aggregates under 3-D RWV culture conditions of 3A6 cells alone (3A6/3A6) or mixed with LNCaP (3A6/LNCaP), C4-2 (3A6/C4-2) or PC3 (3A6/PC3) cells. (C) Detection of mixed cellular components in the 3-D cultured prostate tumoroids by H&E and immunohistochemical staining of E-cadherin (E-Cad) in the cell aggregate sections.

In agreement with several previous reports that MSCs possess intrinsic preferential migratory ability to some epithelial tumors, we demonstrated the migration capacity of human bone marrow-derived MSCs toward prostate cancer cells but not normal prostate epithelial cells in an *in vitro* coculture cell model (Fig. 1A). To further understand whether the recruited human bone marrow-derived MSCs “co-evolve” with prostate cancer cells at the tumor microenvironment, an immortalized human MSC cell line 3A6 [32] was cultured alone or with androgen-dependent LNCaP, its androgen-independent C4-2 subline, or bone metastatic PC3 prostate cancer cells in previously established 3-D RWV culture conditions, recapitulating the *in vivo* tumor microenvironment [13]. We found that cells, either in monoculture or in coculture conditions, formed 3-D ball-like aggregates spontaneously (Fig. 1B). Notably, while 3A6 coculturing with LNCaP (3A6/LNCaP) or C4-2 cells (3A6/C4-2) formed larger aggregates than the 3A6 monoculture (3A6/3A6), a markedly reduced number and size of aggregates were observed in the cultivating group of 3A6 and PC3 cells (3A6/PC3). To identify the cell types that are present in the aggregates, some of the cellular aggregates were subjected to histomorphological analysis. Intense staining of the chromatin-rich cell nucleus (Fig. 1C; H&E) and positive immunoreactivity of the epithelial cell marker E-cadherin (Fig. 1C; IHC) were detected in aggregates harvested from 3A6/LNCaP, 3A6/C4-2 and 3A6/PC3 culture groups but not in aggregates of 3A6 cells grown alone. This finding confirmed the 3-D growth of chimeric tumoroids comprised of prostate cancer cells and MSCs under these coculture conditions.

**Figure 2:**
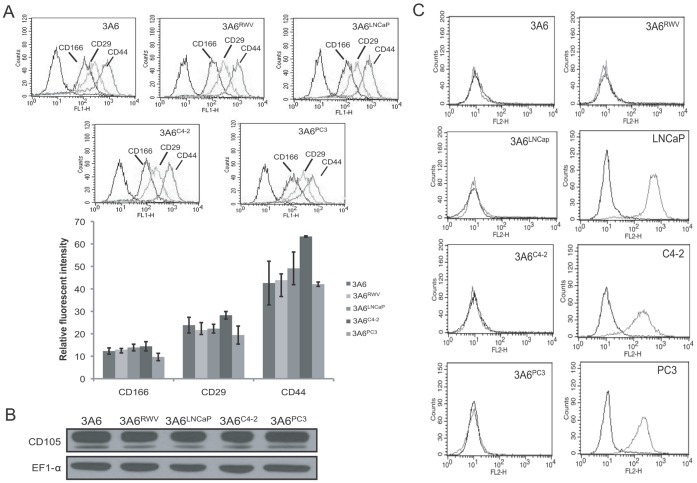
Characterization of *in vitro* established normal and prostate cancer-associated MSC cell lines. (A) Flow cytometric analysis of cell surface antigens of parental 3A6 and its derivatives isolated from 3-D RWV cultured cell aggregates. The histograms display the fluorescence profiles of 3A6 derivatives stained with antibodies against the indicated antigens and the respective isotype control antibody (black line). The expression levels of individual antigens were quantified as the ratio of the mean channel fluorescence of the control antibody and specific antibody. Error bars indicate SD of triplicate measurements. (B) Immunoblotting analysis of CD105 expression in the 3A6 derivatives. EF1-α protein levels are shown to vary in loading quantities. (C) Flow cytometric analysis of the epithelial marker E-cadherin in 3A6 derivatives and their corresponding coculture partners of prostate cancer cell lines. Black lines represent isotype controls.

We further isolated the 3A6 cells from monoculture aggregates and each of the chimeric prostate tumoroids (3A6/LNCaP, 3A6/C4-2 and 3A6/PC3). The resultant 3A6 derivatives that represent the genetically relevant normal MSC and prostate cancer-associated MSC cell lines were designated as 3A6^RWV^ and 3A6^LNCaP^, 3A6^C4-2^ and 3A6^PC3^, respectively. The purity of these 3A6 derivatives was confirmed by assessing MSC surface markers, including CD105, CD166, CD44 and CD29. No significant differences in expression levels were found compared to the parental 3A6 cells that were grown in plastic dishes among all 15 passages (Figs. 2A, 2B and Table S2). The absence of contaminating prostate cancer cells was also demonstrated by the lack of E-cadherin expressing cells (Fig. 2C). These paired normal and prostate cancer-associated MSC cell lines were used for further characterization.

### Prostate Cancer Influences the Lineage Commitment of Bone Barrow-derived MSCs After Contact Coculture

**Figure 3:**
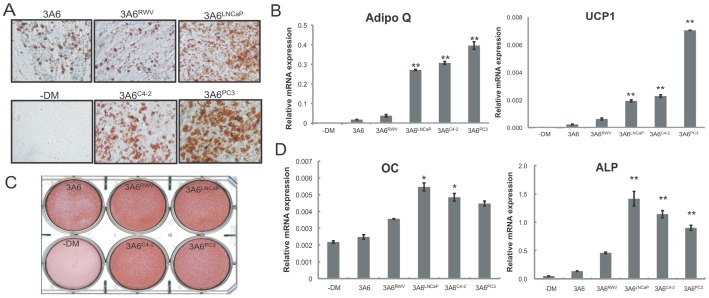
*In vitro* mesenchymal differentiation of normal and prostate cancer-associated MSC cell lines. 3A6 derivatives were cultured in osteogenic or adipogenic medium to assess their multilineage differentiation capability. Parental 3A6 cells grown in the absence of differentiation medium (−DM) were used as the non-induced control. Representative staining of (A) Oil red O staining to determine lipid droplet content after 21 days of differentiation and (C) Alizarin red S to detect bone mineralization 14 days after culture in osteogenic-induction media. Magnification: 100x. (B) qRT-PCR analysis of the expression of common adipogenesis markers and (D) osteoblast marker genes. Values are expressed as normalized mRNA quantities relative to HSPCB housekeeping gene, and error bars represent SD of three independent experiments. *P<0.05; **P<0.001 between normal 3A6^RWV^ and cancer-associated 3A6 derivatives.

We first investigated whether prostate cancer has the potential to direct the differentiation fates of bone marrow-derived MSC after long-term physical contact. We found that 3-D cultured 3A6 derivatives, regardless of whether they were isolated from homotypic cell aggregates or chimeric prostate tumoroids, maintained MSC properties and were able to differentiate into the osteoblasts and adipocytes upon culture in the appropriate differentiation media (Fig. 3). However, an increased tendency toward adipogenic differentiation, which was indicated by enhanced lipid accumulation (Fig. 3A, Fig. S1A) and higher expression of adipocyte-specific markers adiponectin (Adipoq) and uncoupling protein 1 (UCP1) (Fig. 3B) was found in all three cancer-associated 3A6 derivatives with supreme induction seen in 3A6^PC3^ when compared with normal 3A6^RWV^ or parental 3A6 cells. On the other hand, although Alizarin red S staining for matrix mineralization (Fig. 3C, Fig. S1B) concomitant with osteoblast-specific genes alkaline phosphatase (ALP) and osteocalcin (OC) (Fig. 3D) expression showed a minor increase in the osteogenic activity of cancer-associated 3A6 derivatives, a reverse trend between osteogenic and adipogenic differentiation was found among these cell lines. There was no significant difference in growth rate between 3A6^RWV^ and cancer-associated 3A6 derivatives (Fig. S2), which can rule out the possibility that the changed differentiation potential is a consequence of the altered proliferation capacity of the cells.

### Cancer-associated MSCs Exert a Higher Ability to Accelerate Prostate Cancer Growth and Progression than Normal MSCs

**Figure 4:**
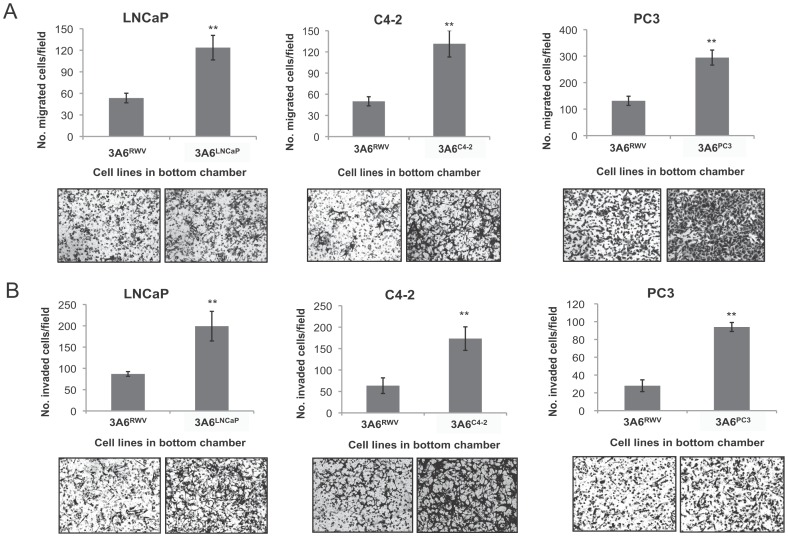
Effects of normal and cancer-associated MSCs on metastatic potentials of prostate cancer cells *in vitro*. Migration (A) and invasion (B) of the indicated prostate cancer cells towards the normal 3A6^RWV^ and the corresponding cancer-associated 3A6 derivatives were analyzed by transwell assay after 8 h and 20 h of incubation, respectively. Data are represented as the means ± SD of the number of cells per high-power field (100x) in triplicate experiments. **P<0.001. The representative image from each condition is shown at bottom.

We next analyzed and compared the biological effect corresponding to the pro-metastatic potential of normal 3A6 with cancer-associated 3A6 in prostate cancer. The migratory and invasive capacity of LNCaP, C4-2 and PC3 prostate cancer cells in response to cues from the corresponding cancer-associated 3A6^LNCaP^, 3A6^C4-2^ and 3A6^PC3^, respectively, or the normal 3A6^RWV^ cells, was assessed via Boyden chamber assays. As shown in Figure 4, all three cancer-associated 3A6 derivatives exerted significantly higher pro-metastatic effects by promoting prostate cancer cell migration (Fig. 4A) and invasion (Fig. 4B) with an average 2- and 3-fold increase in the number of cells moving toward the bottom chamber, respectively, when compared to that of normal 3A6^RWV^ cells.

**Figure 5:**
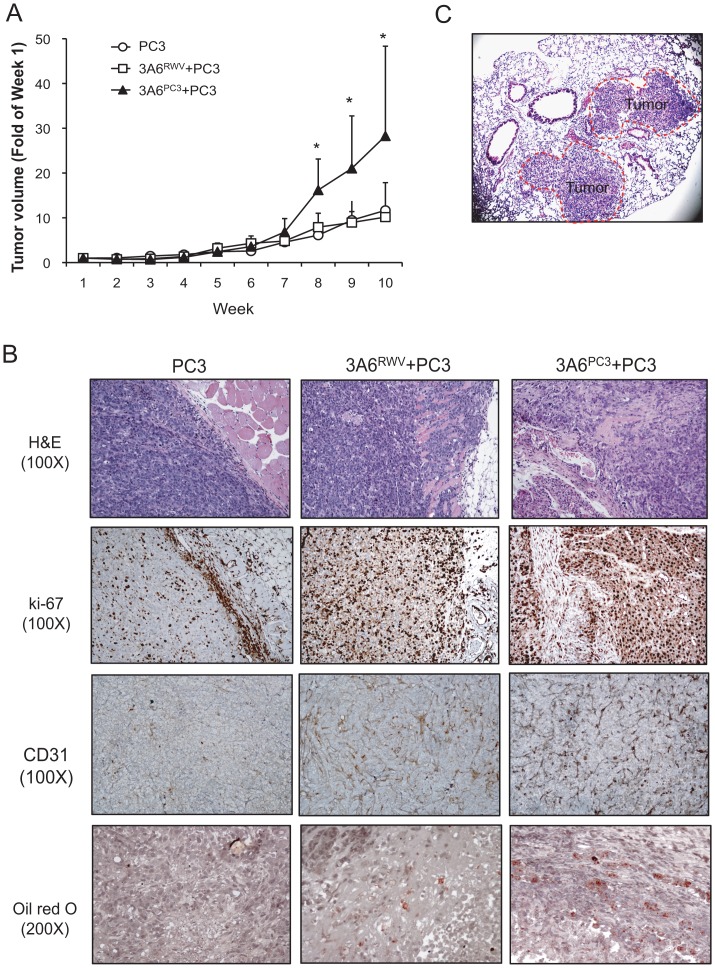
Pro-metastatic effects of normal and cancer-associated MSCs on prostate cancer cells in a xenograft mouse model. Nude mice were implanted subcutaneously with PC3 prostate cancer cells, either alone (PC3) or mixed with normal 3A6^RWV^ (PC3+3A6^RWV^) or cancer-associated 3A6^PC3^ cells (PC3+3A6^PC3^) on the flank (*n = *10 for each group). (A) Tumor growth kinetics were measured over 10 weeks of follow-up and are presented as the fold changes compared to week 1 after cell implantation. *p<0.05 versus PC3 control group. (B) Representative panel of the histological H&E staining, immunohistochemical staining for the cell proliferation marker ki-67 and vascular marker CD31, and Oil red O staining for lipid vacuoles (red) of subcutaneous tumor sections. (C) Lung metastases observed by histological H&E staining of tissue sections (40x) from 3A6^PC3^ co-inoculated PC3 animals. Tumor area was circled and indicated.

To further validate the advanced tumor-promoting behavior of cancer-associated MSCs on prostate cancer *in vivo*, we subcutaneously injected PC3 cells alone or mixed with either normal 3A6^RWV^ (3A6^RWV^/PC3) or cancer-associated 3A6^PC3^ cells (3A6^PC3^/PC3) into nude mice and assessed the impact of 3A6 cells on PC3 tumors. In a parallel study, 3A6^RWV^ and 3A6^PC3^ cells were injected alone, and the result confirmed the nontumorigenic property of 3A6 derivatives (data not shown). Although both 3A6^RWV^ and 3A6^PC3^ had no effect on PC3 cell growth *in vitro* (Fig. S3), a significant increase in tumor volume was observed in 3A6^PC3^/PC3 but not 3A6^RWV^/PC3 xenografts (Fig. 5A). The majority of PC3 tumors in the absence of 3A6 remained circumscribed, whereas PC3 tumors grown together with 3A6^RWV^ or 3A6^PC3^ displayed an infiltrative growth pattern, with invasion into the surrounding stroma and underlying muscle (Fig. 5B, H&E). The highly proliferative and invasive tumor cells were confirmed by ki-67 expression and were associated with high microvessel density (Fig. 5B, CD31). These invasive characteristics are most obviously seen in the 3A6^PC3^/PC3 xenografts. Notably, 1 of 10 mice receiving the 3A6^PC3^/PC3 xenografts developed lung metastases (Fig. 5C), while no metastases were seen in either the group with tumors from PC3 cells alone or those with PC3 cells mixed with normal 3A6^RWV^ cells. In addition, consistent with the *in vitro* finding that cancer-associated 3A6 derivatives have higher adipogenic differentiation potential than normal 3A6^RWV^, Oil red O staining revealed an increased amount of lipid accumulation in the tumor sections of 3A6^PC3^/PC3 compared to that of 3A6^RWV^/PC3 (Fig. 5B, Oil red O).

### IL-6 Contributes to the Reactive Stromal Phenotype Of Cancer-associated MSCs

**Figure 6:**
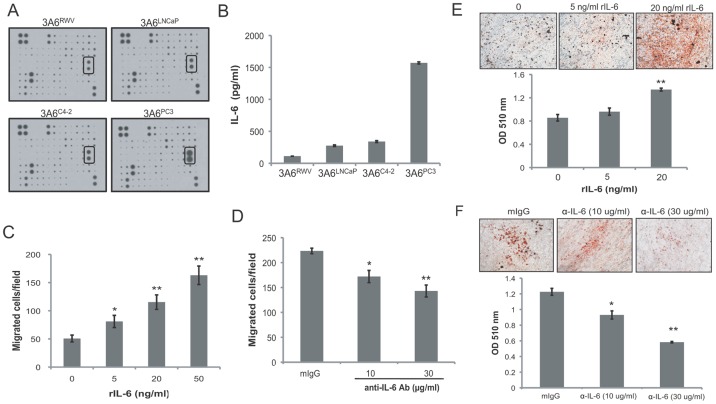
Effects of IL-6 on the reactive stromal phenotypes of MSCs. (A) Cytokine expression profile of the conditioned medium obtained from the normal 3A6^RWV^ and the prostate cancer-associated 3A6^LNCaP^, 3A6^C4-2^, and 3A6^PC3^ was analyzed by using a Human Cytokine Antibody Array C Series 2000. The autoradiograph of one set of Array VI containing IL-6 spots (rectangular boxes) is presented. (B) Quantitative detection of the expression level of IL-6 by ELISA assay. Data represent the means ± SD of triplicate determination. (C) Chemotactic response of PC3 cells induced by human recombinant IL-6 (rIL-6). Data are represented as the means ± SD of the number of cells per high-power field (100x) in triplicate experiments. *P<0.05, **P<0.001 *vs.* serum-free medium alone. (D) Inhibition of PC3 cell migration toward 3A6^PC3^ conditioned medium by the anti-IL-6 antibody. Conditioned medium from 3A6^PC3^ cells were pre-treated with the indicated concentration of mouse anti-IL-6 antibody and then loaded at the bottom chamber of transwells. Treatment with 30 µg/ml of mouse IgG (mIgG) was a negative control. *P<0.05, **P<0.001 *vs.* mouse IgG. (E) Induction of adipogenesis of 3A6^RWV^ cells by human recombinant IL-6, and (F) inhibition of adipogenesis of 3A6^PC3^ cells by anti-IL-6 antibody at the indicated concentration. Cells were stained with Oil red O to visualize lipid droplets after 21 days incubation. The representative image (100x) of each condition is shown at top. The stained cells were quantified using a dye extraction method and data are represented as the means ± SD of three independent experiments. **P<0.001 *vs.* mouse IgG control group.

To identify candidate factors responsible for the reactive stromal activities of cancer-associated MSCs, human cytokine antibody arrays were used to compare the cytokine expression profiles of conditioned medium collected from normal 3A6^RWV^ and cancer-associated 3A6^LNCaP^, 3A6^C4-2^ and 3A6^PC3^ cells. Among 174 cytokines were tested, IL-6, was significantly higher in all three of the cancer-associated 3A6 derivatives compared with 3A6^RWV^ (Fig. 6A). Quantitative data obtained from an ELISA assay (Fig. 6B) confirmed the elevated IL-6 protein levels in 3A6^LNCaP^, 3A6^C4-2^ and 3A6^PC3^ cells corresponding to increases of 145%, 204% and 1403%, respectively, above that of 3A6^RWV^ (112 pg/ml).

We examined whether IL-6 is involved in the induction of prostate cancer cell migration by cancer-associated 3A6. We found that exogenous human IL-6 (rIL-6) induced a dose-dependent increasing in the transwell migration of PC3 cells (Fig. 6C). In addition, the number of PC3 cells that migrated toward the 3A6^PC3^ conditioned medium in the bottom chamber was inhibited by 23% and 36% when the conditioned medium was pre-treated with an IL-6 neutralizing antibody at concentrations of 10 µg/ml and 30 µg/ml, respectively (Fig. 6D).

The role of IL-6 in the enhanced adipogenesis of cancer-associated MSCs was also assessed. Normal 3A6^RWV^ cells were induced to differentiate into adipogenic cells with or without additional rIL-6. Oil red O staining revealed both a qualitative and quantitative increase in lipid droplet formation in 3A6^RWV^ cells under adipogenic differentiation conditions in a rIL-6 dose-dependent manner (Fig. 6E). Conversely, the functional blocking of IL-6 with an anti-IL-6 antibody significantly prevented the differentiation of cancer-associated 3A6^PC3^ into adipocytes (Fig. 6F).

### Let-7 microRNA (miRNA) is Downregulated in Cancer-associated MSCs and Targets IL-6 mRNA

**Figure 7:**
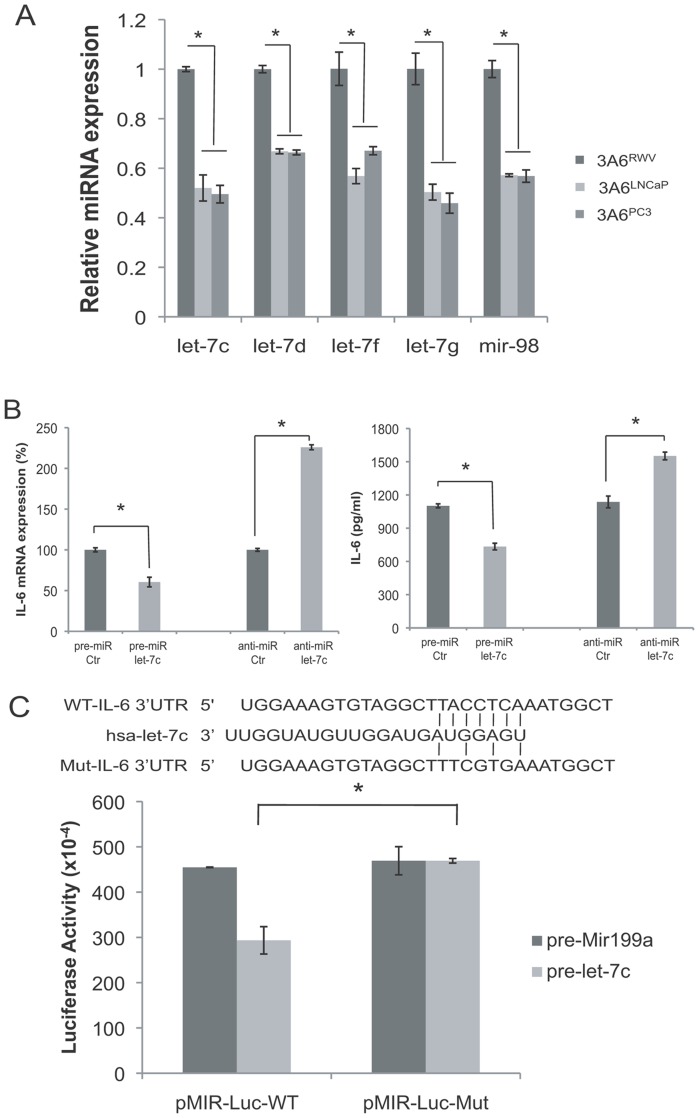
Identification of let-7 as an IL-6 targeting miRNA in cancer-associated MSCs. (A) Quantitative RT-PCR analysis of the expression levels of let-7 miRNA in normal 3A6^RWV^ and cancer-associated 3A6^LNCaP^ and 3A6^PC3^ cell lines. Values are presented as the means ± SD of relative expression levels compared to the U6 internal control and normalized to the 3A6^RWV^ cells of each matched pair. *P<0.005. (B) Regulation of IL-6 by let-7. 3A6 cells were transfected with the let-7c precursor (pre-miR let-7c) or let-7 family inhibitors (anti-miR let-7). Cells and conditioned medium were harvested 72 h after transfection, and IL-6 RNA and protein levels were determined by qRT-PCR and ELISA, respectively. *P<0.005. (C) IL-6 response element reporter assay with let-7c precursor or let-7 family inhibitors. The seed region of the let-7c potential target sites in human IL-6 was predicted by microRNA.org. Top, wild-type IL-6 sequence; middle, let-7c sequence; bottom, mutant IL-6 sequence. 3′-UTR vectors with wild-type (pMIR-Luc-WT) or mutant IL-6 (pMIR-Luc-Mut) sequence were cotransfected with let-7c or Mir-199a precursors into HEK 293 cells. Luciferase activities were assayed 48 h after transfection and normalized to internal control β-galactosidase activity. Data are the means ± SD of three independent experiments, *P<0.05.

MicroRNAs (miRNAs) are characterized as gene silencers that negatively regulate gene expression. We, therefore, investigated whether the overexpression of IL-6 by cancer-associated MSCs occurs through the regulation of miRNAs. Among 9 miRNAs that were characterized to be significantly downregulated in both 3A6^LNCaP^ and 3A6^PC3^ cells in comparison with 3A6^RWV^ by microarray analysis (data not shown), let-7c, let-7d, let-7g, let-7f, and miR-98 are members of the let-7 family and were identified to potentially bind the 3′-UTR of IL-6 mRNA using the TargetScan algorithm. Quantitative RT-PCR results confirmed the significantly decreased expression of these let-7 family members in 3A6^LNCaP^ and 3A6^PC3^ cells compared to normal 3A6^RWV^ (Fig. 7A).

To determine whether the let-7 miRNA is a critical mediator regulating IL-6 expression in MSCs, we overexpressed let-7 in 3A6 cells by transfecting cells with a synthetic let-7c precursor as the representative let-7 miRNA because of its higher expression than other members in 3A6 (data not shown), or inhibited expression levels using anti-miR oligonucleotides complementary to mature let-7 sequences, respectively. Quantitative RT-PCR and ELISA analysis showed that IL-6 expression was decreased in let-7c precursor-transfected cells and increased in anti-miR-let-7-transfected cells compared with the respective control oligonucleotides (Fig. 7B). In addition, a luciferase reporter assay revealed a significant reduction of luciferase activity in 3A6 cells that were transfected with the pMir-Luc reporter-containing wild-type but not the mutant form of IL-6 3′-UTR (the predicted site for let-7c binding) upon let-7c precursor cotransfection (Fig. 7C). Moreover, cotransfection of these reporter vectors with a let-7 unrelated miRNA, miR-199a, caused no difference in luciferase activity, further confirming the specificity of the binding sequences. Taken together, these results demonstrated that IL-6 is the direct target of let-7 in MSCs.

### Let-7 miRNA Inhibits Reactive Stromal Phenotypes of Cancer-associated MSCs

**Figure 8:**
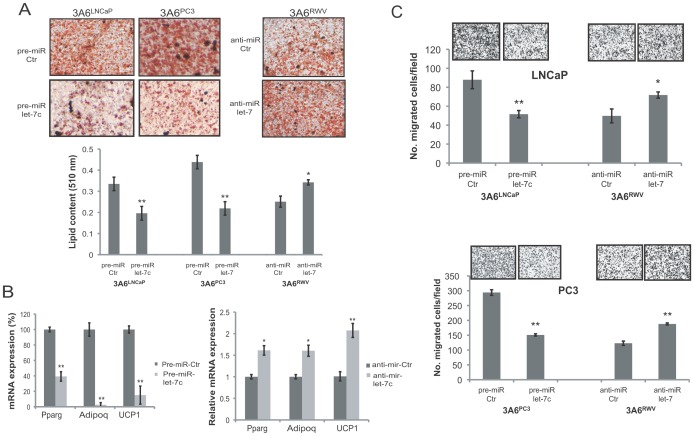
Effect of let-7 on reactive phenotypes of MSCs. Cancer-associated 3A6^LNCaP^ and 3A6^PC3^ cells transfected with the let-7c precursor or miR control, and normal 3A6^RWV^ cells transfected with let-7 inhibitors or the anti-miR control were induced to adipogenic differentiation (A, B), or were cocultured with prostate cancer cell lines for transwell migration assay (C). (A) Representative micrographs of the Oil red O-stained adipocytes from transfected 3A6 derivatives 21 days after the induction of differentiation (100x). The quantification of Oil red O staining was performed by dye extraction and measuring the absorbance at 510 nm. (B) Quantitative RT-PCR was carried out to detect the expression of adipocyte markers. (C) LNCaP and PC3 prostate cancer cells that migrated toward the indicated 3A6 derivatives in the lower chamber of transwells were stained with crystal violet 12 h after incubation and then photographed (100x, top). Data are represented as the means ± SD of the number of cells per high-power field (100x) in triplicate experiments. ^*^ *P*<0.05; ^**^ *P*<0.005 *vs.* control.

To characterize the biological effects of downregulated let-7 in the behavioral changes of cancer-associated MSCs, we modulated the expression levels of let-7 in the normal 3A6^RWV^ and the cancer-associated 3A6^LNCaP^ and 3A6^PC3^ cells by transient transfection of the anti-miR let-7 and let-7c precursor, respectively (Fig. S4). The impact of altered let-7 expression on adipogenic differentiation was first determined. Oil red O staining demonstrated that the high content of lipid droplets in 3A6^LNCaP^ and 3A6^PC3^ cells was markedly decreased by approximately 50% upon let-7c precursor transfection. Conversely, the inhibition of let-7 expression in normal 3A6^RWV^ cells resulted in a 20% increased level of adipocyte formation (Fig. 8A). This result corresponded to our marker analysis in which the expression of PPARγ, Adipoq, and UCP1 was suppressed in 3A6^LNCaP^ and 3A6^PC3^ cells when the cells were transfected with the let-7c precursor and upregulated in anti-miR let-7-treated 3A6^RWV^ cells compared to the control oligonucleotides-transfected cells (Fig. 8B). We next analyzed the effects of let-7 on the pro-metastatic activity of MSCs. We found that the transfection of let-7c into either 3A6^LNCaP^ or 3A6^PC3^ cells dramatically impaired its ability to attract prostate cancer cell migration. Moreover, let-7 blockage in 3A6^RWV^ cells augmented the chemotactic responsiveness of prostate cancer cells *in vitro* (Fig. 8C). The inhibitory effects of ectopic let-7 expression on adipogenesis and pro-metastatic activity of 3A6^PC3^ cells can be reversed by additional IL-6 (Fig. S5). Collectively, these results demonstrated that let-7 downregulation confers the reactive stromal phenotypes of prostate cancer-associated MSCs through its target gene IL-6.

## Discussion

Although genetic and/or epigenetic alterations in cancer epithelium are expected and widely characterized, little progress has been made in elucidating the molecular changes in the stroma in response to cancer epithelium and their potential functions. This lack of progress is in part due to the lack of a well-defined system by which to study the cellular interactions between relevant cell types *in vivo* and *in vitro*. In the present study, we showed that bone marrow-derived MSCs were able to form homotypic spheroids or heterotypic tumoroids with different prostate cancer cell lines, and these cells are able to maintain their multipotent differentiation potential under dynamic RWV culture conditions. In addition, there are fundamental differences between MSCs isolated from tumoroids and homotypic spheroids with distinct cytokine and microRNA expression profiles, enhanced adipogenic differentiation potential and increased malignancy induction, which are the stromal reactions that are frequently characterized in human prostate cancer [36]. Due to the limited availability of live cells obtained from bone metastasis specimens of prostate cancer, this RWV coculture model mimicking the *in vivo* epithelial-stromal interaction, as we demonstrated previously [13] and herein, represents a simple and reliable experimental model with which to establish the matched pairs of normal and cancer-associated bone stromal cell lines for mechanistic studies of prostate cancer bone metastasis.

Under the 3-D RWV culture conditions, bone marrow-derived MSCs appeared to respond differently with prostate cancer cell lines, which induce distinct characteristics of bone remodeling. LNCaP and C4-2 cells, which have the higher potential to induce osteoblastic lesions with mild effects on bone destruction [37], developed faster and larger tumoroids with MSCs than MSC homotypic spheroids. In contrast, PC3 cells that produce a pure osteolytic reaction in the bone [38] disturbed MSC intracellular adhesions and caused only a small fraction of cells to form heterotypic aggregates. The precise mechanisms that cause the smallest aggregates of MSCs with PC3 than that with LNCaP and C4-2 are not fully understood. Intercelluar adhesion relies heavily on the association between cell adhesion molecules expressed on the cell surfaces. The difference in the expression profiles of cell adhesion molecules among LNCaP, C4-2 and PC3 cells [39] may be the major causes of varied aggregate size seen in the present study. It has been demonstrated that cell shape, which is determined by the internal organization of the cytoskeleton as well as by interactions between adjacent cells and the surrounding matrix, has a strong influence on the switch in lineage commitment of MSCs. For example, MSCs permitted to spread out and flatten is highly osteogenic compared with round cells that instead become adipogenic [40]. Likewise, the unfavored cell-cell contact of MSCs with osteolytic PC3 prostate cancer cells may provide adhesive cues critical for directing the adipogenic differentiation seen in the corresponding 3A6^PC3^ cells. In addition, it has been proposed that the balance between the osteoblast and adipocyte lineages is tilted toward increased adipocytes and decreased osteoblasts during age-related osteoporosis, which leads to progressive fatty bone marrow with bone loss [41]. As the similarity of pathogenesis in osteoporosis and osteolytic metastases, the correlation between the switch from osteogenesis to adipogenesis in bone marrow-derived MSCs and the ability of their associated prostate cancer cell lines to induce osteolytic lesions implies that interactions between prostate cancer and MSCs could be a critical determinant of skeletal pathogenesis. Indeed, Zhau *et al* recently revealed abnormally enhanced adiopogenic marker expression in clinical prostate cancer bone metastasis [42]. Although they showed that prostate cancer harbors the MSC property for mesengenic differentiation *in vitro*, our study suggests the possibility that cancer-associated MSCs may be, at least in part, the source of the increased adipocytes within the tumor microenvironment. This study is thus the first to recognize pro-adipogenic function of prostate cancer in bone marrow-derived MSCs.

IL-6 levels have been shown to be frequently elevated in prostate cancer patients, which is correlated with a poor prognosis and bone metastasis of this disease [43], [44]. Experimental data have proposed IL-6 as a growth factor that regulates the proliferation [45]–[47] and survival [48], [49] of prostate cancer cells. In addition to this known pro-tumorigenic activity, our data uncovered a pro-metastatic role of paracrine IL-6 in the tumor microenvironment, which facilitates cancer cell migration and invasion. The precise mechanisms that promote prostate cancer metastasis by environmental IL-6 are not fully understood. A recent study has suggested that IL-6 can induce an invasive phenotype in non-tumorigenic prostate epithelial cells through epithelial-to-mesenchymal transition (EMT) [50]. In the models of breast cancer [51], head and neck cancer [52] and lung cancer [53], IL-6 also serves as an inducer of EMT. Given the growing evidence that EMT states exist and may contribute to prostate cancer progression and metastasis, it is therefore not surprising that IL-6 signaling is perhaps involved in the processes of prostate cancer-associated EMT during their metastatic dissemination. Intriguingly, some data suggest that the interaction of MSCs and colorectal cancer cells stimulates the production of IL-6 by MSCs, which induces cancer stem cell formation [54] and recruits endothelial cells to the tumor sites [55]. This type of interaction may explain why the PC3 tumor growth enhancement by cancer-associated MSCs seen in experimental animals was not observed in the coculture cell model *in vitro*. In fact, a strong intensity as well as a high percentage of CD31 staining was found in tumor sections where PC3 cancer cells were co-inoculated with MSCs, in particular with cancer-associated 3A6^PC3^, suggesting an inductive role of prostate cancer cells in MSC-mediated vasculogenesis.

Several miRNAs and their target genes have been identified to regulate adipogenesis in MSCs [56], [57]. Unlike these miRNAs are upregulated, we have shown here that the loss of let-7 in bone marrow-derived MSCs triggers their adipogenic differentiation through upregulating IL-6 expression. Other than let-7 family members that were significantly downregulated in both 3A6^LNCaP^ and 3A6^PC3^ in comparison with 3A6^RWV^, some miRNAs were found up or down only in one cell line by microarray analysis. There is a possibility that these 3A6^LNCaP^-specific miRNAs and/or -associated genes involve in the regulation of IL-6 expression by pass the function of let-7, and this could explain, at least in part, why 3A6^LNCaP^ and 3A6^PC3^ expressed similar level of let-7 but different amount of IL-6. However, the causative molecular basis of let-7 alterations in cancer-associated MSCs is not yet understood. A recent study [58] reported a regulatory circuit in which inflammatory signals stimulate NF-κB nuclear translocation which directly activates LIN28 transcription and substantially inhibits the biogenesis of let-7, thereby generating high levels of IL-6, an activator of NF-κB. Prostate cancer cell lines are known to produce varying amounts of IL-6 [59]. Thus, it is possible that one of the reasons for the downregulation of let-7 in cancer-associated MSCs is increased LIN28 activity through the positive feedback loop of IL-6 that is initiated by prostate cancer cells. Importantly, repressed let-7 expression was observed in various cancers [60], including prostate cancer [61], and contributes to carcinoma aggressiveness. Treatment with exogenous let-7 would target both cancer cells and their associated MSCs and could be an attractive therapeutic approach to effectively inhibit prostate cancer recurrence and prevent metastasis.

In summary, we have provided the first evidence that prostate cancer-associated MSCs exhibit greater potential to augment prostate cancer metastasis in comparison with normal MSCs. The autocrine production of IL-6 through the downregulation of let-7 miRNA by MSCs, in particular those that are associated with osteolytic prostate cancer, is central to facilitating the adipogenic differentiation of MSCs and for their supporting effects on cancer metastasis. This report is thus the first to define the mesenchymal switch and epigenetic changes of prostate cancer-associated MSCs. Our data also suggest that bone marrow MSCs directly participate in the pathogenesis of prostate cancer bone metastases, which provides an alternative and important pathway by which prostate cancer cells can regulate bone remodeling. Further investigation of how osteolytic and osteoblastic cancer cells influence the adipogenic-osteogenic switch of MSCs may lead to new therapeutic avenues for an incurable disease.

## Supporting Information

Figure S1 **Quantification of adipogenesis and osteogenesis of 3A6 derivatives.** (A) Oil red O staining to determine lipid droplet content after 21 days of differentiation and (B) Alizarin red S to detect calcium content 14 days after culture in osteogenic-induction media. The stained cells were quantified using a dye extraction method and data are represented as the means ± SD. *P<0.05; **P<0.001 between normal 3A6^RWV^ and cancer-associated 3A6 derivatives.
(PDF)

Figure S2 **Comparison of cell proliferation among 3A6 derivatives by WST-1 assay performed daily for 5 days.** The relative cell number was assessed by absorbance at 450 nm and presented as the fold change relative to the day of plating (day 0). Error bars indicate SD of triplicate measurements. NS = not significant.
(PDF)

Figure S3 **PC3 cell proliferation under the coculture with 3A6 derivatives.** Luciferase-expressing PC3 cells were seeded alone (control) or mixed with equal number of 3A6^RWV^ or 3A6^PC3^ cells in 6-well plates. PC3 cell proliferation was determined by luciferase activity at indicated time points. Data were plotted as relative luciferase unit (RLU).
(PDF)

Figure S4 **Expression level of let-7 in the transfectants of 3A6 derivatives.** The 3A6^RWV^ normal MSCs (A) and the 3A6^LNCaP^ and 3A6^PC3^ cancer-associated MSCs (B) were transfected with the indicated anti-miR and miRNA precursor for 72 hr, respectively, and then subjected to quantitative RT-PCR analysis for the expression of let-7c. Values are presented as the means ± SD of relative expression levels of let-7c expression in the let-7-specific transfectants compared to that of negative control (Ctr) oligonucleotide transfectants after normalized with the U6 internal control.
(PDF)

Figure S5 **Reverse effects of IL-6 on exogenous let-7c-suppressed reactive phenotypes of MSCs.** Cancer-associated 3A6^PC3^ cells transfected with let-7c precursor were induced to adipogenic differentiation or were cocultured with PC3 prostate cancer cells for transwell migration and invasion assay with the indicated concentration of recombinant IL-6. A microRNA transfection control (pre-mir-Ctr) was used to determine the basal activity of 3A6^PC3^. Representative images of Oil red O staining for adipogenesis and crystal violet staining for transwell migration and invasion from each condition are shown at top. The quantitative data represented as the means ± SD for triplicate incubations are shown at the bottom. *P<0.05; **P<0.001.
(PDF)

Table S1 **List of primers and probes used in quantitative PCR.**
(PDF)

Table S2 **Immunophenotype of MSC cell lines derived from 3-D RWV co-culture system at different passages.**
(PDF)
