# Supplementary material for: Loss of Let-7 MicroRNA Upregulates IL-6 in Bone Marrow-Derived Mesenchymal Stem Cells Triggering a Reactive Stromal Response to Prostate Cancer
Source: PLoS One. 2013 Aug 19;8(8):e71637. doi: 10.1371/journal.pone.0071637 (PMC3747243; doi:10.1371/journal.pone.0071637)
Supplement: Figure S1 — Quantification of adipogenesis and osteogenesis of 3A6 derivatives. (A) Oil red O staining to determine lipid droplet content after 21 days of differentiation and (B) Alizarin red S to detect calcium content 14 days after culture in osteogenic-induction media. The stained cells were quantified using a dye extraction method and data are represented as the means ± SD. *P<0.05; **P<0.001 between normal 3A6RWV and cancer-associated 3A6 derivatives. (PDF) [file pone.0071637.s001.pdf]

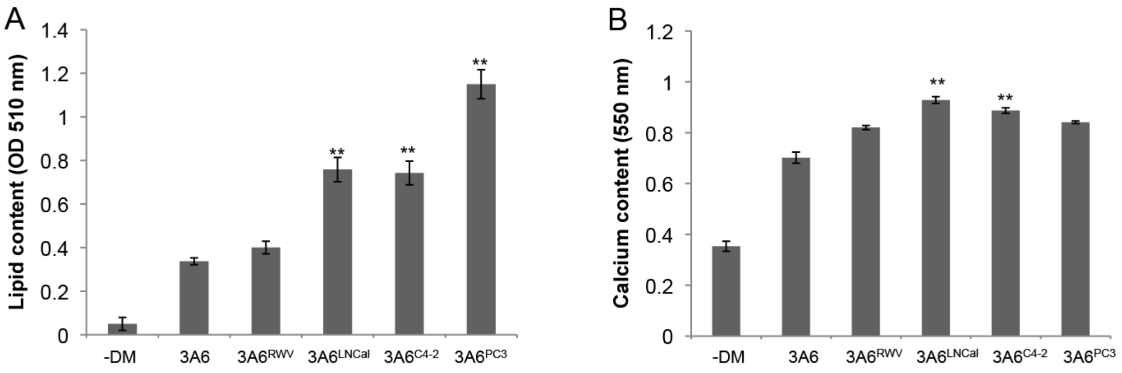

Supplementary Figure S1. Quantification of adipogenesis and osteogenesis of 3A6 derivatives. (A) Oil red O staining to determine lipid droplet content after 21 days of differentiation and (B) Alizarin red S to detect calcium content 14 days after culture in osteogenic-induction media. The stained cells were quantified using a dye extraction method and data are represented as the means ± SD. $^{**}P < 0.001$ between normal $3A6^{RWV}$ and cancer-associated 3A6 derivatives.
