# Supplementary material for: Loss of Let-7 MicroRNA Upregulates IL-6 in Bone Marrow-Derived Mesenchymal Stem Cells Triggering a Reactive Stromal Response to Prostate Cancer
Source: PLoS One. 2013 Aug 19;8(8):e71637. doi: 10.1371/journal.pone.0071637 (PMC3747243; doi:10.1371/journal.pone.0071637)
Supplement: Figure S2 — Comparison of cell proliferation among 3A6 derivatives by WST-1 assay performed daily for 5 days. The relative cell number was assessed by absorbance at 450 nm and presented as the fold change relative to the day of plating (day 0). Error bars indicate SD of triplicate measurements. NS = not significant. (PDF) [file pone.0071637.s002.pdf]

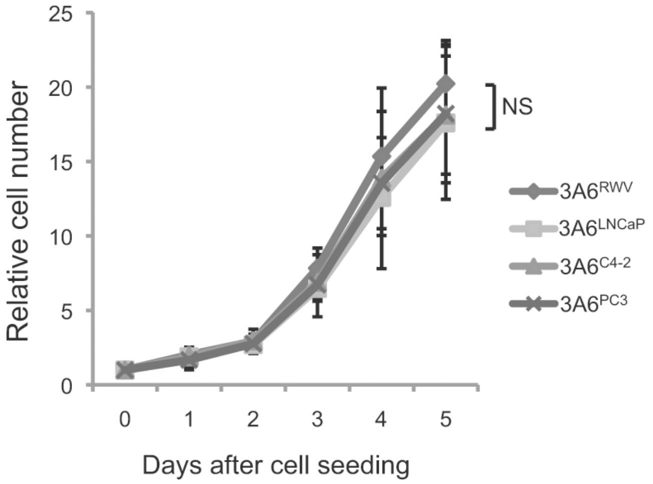

Supplementary Figure S2. Comparison of cell proliferation among 3A6 derivatives by WST-1 assay performed daily for 5 days. The relative cell number was assessed by absorbance at 450 nm and presented as the fold change relative to the day of plating (day 0). Error bars indicate SD of triplicate measurements. NS=not significant.
