# Supplementary material for: Loss of Let-7 MicroRNA Upregulates IL-6 in Bone Marrow-Derived Mesenchymal Stem Cells Triggering a Reactive Stromal Response to Prostate Cancer
Source: PLoS One. 2013 Aug 19;8(8):e71637. doi: 10.1371/journal.pone.0071637 (PMC3747243; doi:10.1371/journal.pone.0071637)
Supplement: Figure S3 — PC3 cell proliferation under the coculture with 3A6 derivatives. Luciferase-expressing PC3 cells were seeded alone (control) or mixed with equal number of 3A6RWV or 3A6PC3 cells in 6-well plates. PC3 cell proliferation was determined by luciferase activity at indicated time points. Data were plotted as relative luciferase unit (RLU). (PDF) [file pone.0071637.s003.pdf]

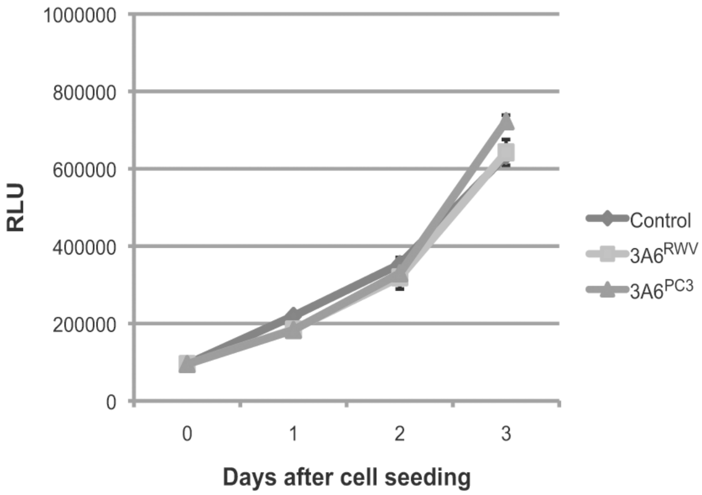

Supplementary Figure S3. PC3 cell proliferation under the coculture with 3A6 derivatives. Luciferase-expressing PC3 cells were seeded alone (control) or mixed with equal number of 3A6RWV or 3A6PC3 cells in 6-well plates. PC3 cell proliferation was determined by luciferase activity at indicated time points. Data were plotted as relative luciferase unit (RLU).
