# Supplementary material for: Loss of Let-7 MicroRNA Upregulates IL-6 in Bone Marrow-Derived Mesenchymal Stem Cells Triggering a Reactive Stromal Response to Prostate Cancer
Source: PLoS One. 2013 Aug 19;8(8):e71637. doi: 10.1371/journal.pone.0071637 (PMC3747243; doi:10.1371/journal.pone.0071637)
Supplement: Figure S4 — Expression level of let-7 in the transfectants of 3A6 derivatives. The 3A6RWV normal MSCs (A) and the 3A6LNCaP and 3A6PC3 cancer-associated MSCs (B) were transfected with the indicated anti-miR and miRNA precursor for 72 hr, respectively, and then subjected to quantitative RT-PCR analysis for the expression of let-7c. Values are presented as the means ± SD of relative expression levels of let-7c expression in the let-7-specific transfectants compared to that of negative control (Ctr) oligonucleotide transfectants after normalized with the U6 internal control. (PDF) [file pone.0071637.s004.pdf]

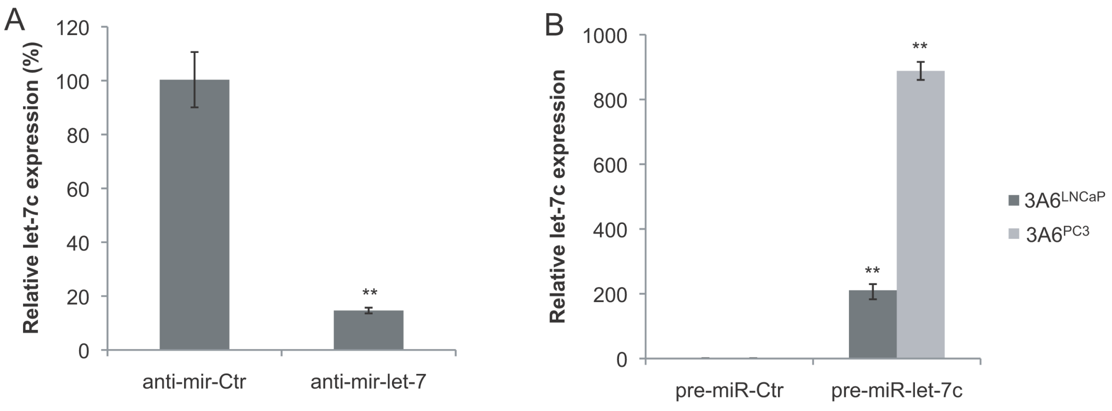

Supplementary Figure S4. Expression level of let-7 in the transfectants of 3A6 derivatives. The 3A6^{RWV} normal MSCs (A) and the 3A6^{LNCaP} and 3A6^{PC3} cancer-associated MSCs (B) were transfected with the indicated anti-miR and miRNA precursor for 72 hr, respectively, and then subjected to quantitative RT-PCR analysis for the expression of let-7c. Values are presented as the means ± SD of relative expression levels of let-7c expression in the let-7-specific transfectants compared to that of negative control (Ctr) oligonucleotide transfectants after normalized with the U6 internal control. **P<0.005.
