# Supplementary material for: Loss of Let-7 MicroRNA Upregulates IL-6 in Bone Marrow-Derived Mesenchymal Stem Cells Triggering a Reactive Stromal Response to Prostate Cancer
Source: PLoS One. 2013 Aug 19;8(8):e71637. doi: 10.1371/journal.pone.0071637 (PMC3747243; doi:10.1371/journal.pone.0071637)
Supplement: Figure S5 — Reverse effects of IL-6 on exogenous let-7c-suppressed reactive phenotypes of MSCs. Cancer-associated 3A6PC3 cells transfected with let-7c precursor were induced to adipogenic differentiation or were cocultured with PC3 prostate cancer cells for transwell migration and invasion assay with the indicated concentration of recombinant IL-6. A microRNA transfection control (pre-mir-Ctr) was used to determine the basal activity of 3A6PC3. Representative images of Oil red O staining for adipogenesis and crystal violet staining for transwell migration and invasion from each condition are shown at top. The quantitative data represented as the means ± SD for triplicate incubations are shown at the bottom. *P<0.05; **P<0.001. (PDF) [file pone.0071637.s005.pdf]

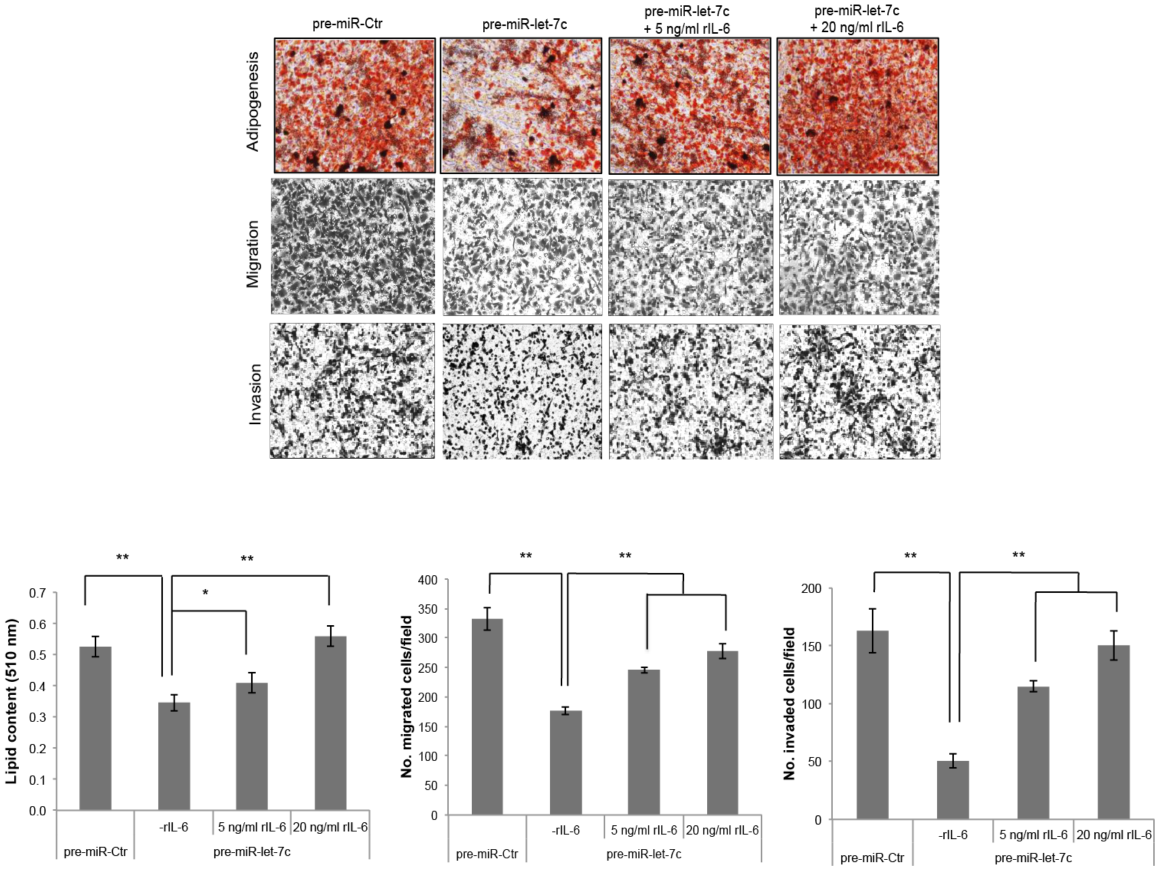

Supplementary Figure S5. Reverse effects of IL-6 on exogenous let-7c-suppressed reactive phenotypes of MSCs. Cancer-associated $3A6^{PC3}$ cells transfected with let-7c precursor were induced to adipogenic differentiation or were cocultured with PC3 prostate cancer cells for transwell migration and invasion assay with the indicated concentration of recombinant IL-6. A microRNA transfection control (pre-mir-Ctr) was used to determine the basal activity of $3A6^{PC3}$. Representative images of Oil red O staining for adipogenesis and crystal violet staining for transwell migration and invasion from each condition are shown at top. The quantitative data represented as the means ± SD for triplicate incubations are shown at the bottom. *$P < 0.05$; **$P < 0.001$.
