# Supplementary material for: Loss of Let-7 MicroRNA Upregulates IL-6 in Bone Marrow-Derived Mesenchymal Stem Cells Triggering a Reactive Stromal Response to Prostate Cancer
Source: PLoS One. 2013 Aug 19;8(8):e71637. doi: 10.1371/journal.pone.0071637 (PMC3747243; doi:10.1371/journal.pone.0071637)
Supplement: Table S1 — List of primers and probes used in quantitative PCR. (PDF) [file pone.0071637.s006.pdf]

Supplementary Table S1: List of primers and probes used in quantitative PCR

Gene	Primer sequences*	UPL†	miRNA	Primer sequences*	UPL†
IL-6	F: gatgagtacaaaagtcctgatcca<br>R: ctgcagccactggttctgt	#40	let-7c	RT: [stem-loop]-aaccat<br>F: cgggttgaggtagtaggttgt	#21
AdipoQ	F: ggtgagaaaggagatccaggt<br>R: tgctgagcggtatacataggc	#41	let-7d	RT: [stem-loop]-aactat<br>F: cctaggaagaggtagtaggttgc	#21
Pparγ	F: gacaggaaagacaacagacaaat<br>R: ggggtgatgtgtttgaacttg	#7	let-7f	RT: [stem-loop]-aactat<br>F: tgtgggatgaggtagtagattgt	#21
UCP1	F: ctggacacggccaaagtc<br>R: ggacacctttatacctaataacactgg	#47	let-7g	RT: [stem-loop]-aactgt<br>F: aggctgaggtagtagtttgt	#21
OC	F: tgagagccctcacactcctc<br>R: acctttgctggactctgcac	#81	Mir-98	RT: [stem-loop]-aacaat<br>F: ccagggtgaggtagtaagttgt	#21
ALP	F: agaaccccaaaggcttcttc<br>R: cttggcttttccttcatggt	#31	U6	RT: [stem-loop]-aaatat<br>F: ttcctccgcaaggatgacacgc	#21
HSPCB	F: agcctacgttgcactattacg<br>R: gaaaggcaaagtctccacct	#55		U: gtgcagggtccgaggt	

† Universal Probe Library (Roche Applied Science)
* F, Forward; R, Reverse; RT, Reverse Transcription; U, Universal Reverse
[stem-loop]: gttggctctggtgcagggtccgaggtattcgcaccagagccaac
