# Supplementary material for: Loss of Let-7 MicroRNA Upregulates IL-6 in Bone Marrow-Derived Mesenchymal Stem Cells Triggering a Reactive Stromal Response to Prostate Cancer
Source: PLoS One. 2013 Aug 19;8(8):e71637. doi: 10.1371/journal.pone.0071637 (PMC3747243; doi:10.1371/journal.pone.0071637)
Supplement: Table S2 — Immunophenotype of MSC cell lines derived from 3-D RWV co-culture system at different passages. (PDF) [file pone.0071637.s007.pdf]

Supplementary Table S2. Immunophenotype of MSC cell lines derived from 3-D RWV co-culture system at different passages

| | | *Surface markers (relative fluorescent intensity)* | | |
|---|---|---|---|---|
| *Cell lines* | *Passages* | CD166 | CD29 | CD44 |
| $3A6^{RWV}$ | P1 | 12.32 ±0.78 | 26.93 ±0.90 | 50.82 ±4.94 |
| | P6 | 12.97 ±0.53 | 21.63 ±3.36 | 46.85 ±2.83 |
| | P15 | 11.37 ±1.07 | 18.96 ±3.05 | 51.48 ±6.38 |
| $3A6^{LNCaP}$ | P1 | 13.65 ±1.42 | 20.75 ±0.94 | 41.16±5.90 |
| | P6 | 13.88 ±1.50 | 22.28 ±1.95 | 49.15 ±7.27 |
| | P15 | 12.75 ±0.85 | 20.14 ±1.37 | 46.19 ±8.00 |
| $3A6^{C4\text{-}2}$ | P1 | 14.76 ±2.50 | 28.57±2.98 | 57.56 ±7.48 |
| | P6 | 14.41 ±2.07 | 28.24±1.68 | 63.33 ±0.28 |
| | P15 | 14.09±3.80 | 31.18 ±5.23 | 81.27 ±9.16 |
| $3A6^{PC3}$ | P1 | 13.28 ±1.10 | 23.69 ±1.25 | 46.66 ±5.19 |
| | P6 | 9.68 ±1.64 | 19.43 ±4.00 | 42.07 ±0.97 |
| | P15 | 17.64 ±1.66 | 20.56 ±4.84 | 61.83±3.84 |

Data are expressed as mean±standard deviation.
